# A noval noninvasive targeted therapy for osteosarcoma: the combination of LIFU and ultrasound-magnetic-mediated SPIO/TP53/PLGA nanobubble

**DOI:** 10.3389/fbioe.2024.1418903

**Published:** 2024-06-28

**Authors:** Honglei Ren, Shanlin Xiang, Aiguo Liu, Qian Wang, Nian Zhou, Zhenming Hu

**Affiliations:** ^1^ Department of Orthopedic Surgery, The First Affiliated Hospital of Chongqing Medical University, Chongqing, China; ^2^ Department of Orthopedic Surgery, ChongQing Red Cross Hospital (People’s Hospital of JiangBei District), Chongqing, China; ^3^ Department of Orthopedics, Shanghai Pudong Hospital, Fudan University Pudong Medical Center, Shanghai, China; ^4^ Department of Orthopedic Surgery, The First Affiliated Hospital of Henan University, Kaifeng, China; ^5^ Department of Orthopedic Surgery, The University-Town Hospital of Chongqing Medical University, Chongqing, China

**Keywords:** osteosarcoma, low-intensity-focused ultrasound, nanobubbles, superparamagnetic iron oxide, PLGA

## Abstract

**Purpose:**

Osteosarcoma (OS) is the most common type of primary malignant bone tumor. Transducing a functional TP53 gene can effectively inhibit OS cell activity. Poly lactic acid-glycolic acid (PLGA) nanobubbles (NBs) mediated by focused ultrasound (US) can introduce exogenous genes into target cells in animal models, but this technique relies on the passive free diffusion of agents across the body. The inclusion of superparamagnetic iron oxide (SPIO) in microbubbles allows for magnetic-based tissue localization. A low-intensity-focused ultrasound (LIFU) instrument was developed at our institute, and different intensities of LIFU can either disrupt the NBs (RLI-LIFU) or exert cytocidal effects on the target tissues (RHI-LIFU). Based on these data, we performed US-magnetic-mediated TP53-NB destruction and investigated its ability to inhibit OS growth when combined with LIFU both *in vitro* and *in vivo*.

**Methods:**

Several SPIO/TP53/PLGA (STP) NB variants were prepared and characterized. For the *in vitro* experiments, HOS and MG63 cells were randomly assigned into five treatment groups. Cell proliferation and the expression of TP53 were detected by CCK8, qRT-PCR and Western blotting, respectively. *In vivo*, tumor-bearing nude mice were randomly assigned into seven treatment groups. The iron distribution of Perls’ Prussian blue-stained tissue sections was determined by optical microscopy. TUNEL-DAPI was performed to examine apoptosis. TP53 expression was detected by qRT-PCR and immunohistochemistry.

**Results:**

SPIO/TP53/PLGA NBs with a particle size of approximately 200 nm were prepared successfully. For *in vitro* experiments, ultrasound-targeted transfection of TP53 overexpression in OS cells and efficient inhibition of OS proliferation have been demonstrated. Furthermore, in a tumor-bearing nude mouse model, RLI-LIFU-magnetic-mediated SPIO/TP53/PLGA NBs increased the transfection efficiency of the TP53 plasmid, resulting in apoptosis. Adding RHI-LIFU to the treatment regimen significantly increased the apoptosis of OS cells *in vivo*.

**Conclusion:**

Combining LIFU and US-magnetic-mediated SPIO/TP53/PLGA NB destruction is potentially a novel noninvasive and targeted therapy for OS.

## Introduction

Osteosarcoma (OS) is the most common type of primary malignant bone tumor and is characterized by local invasion and distant metastasis. The current clinical treatment modalities for OS include surgical resection of primary tumors and systemic chemotherapy. Research has shown that the application of neoadjuvant chemotherapy increases the tumor-free survival of OS from 1% to 50%–65%. However, resistance of OS cells to chemotherapeutic drugs remains a major limiting factor for current treatment modalities for this disease. Additionally, surgical treatment for OS may cause significant physical trauma. Therefore, a new, less traumatic therapy would be of great significance.

In recent years, gene therapy for OS has emerged as an exciting novel research field. TP53 was found to be the most frequently mutated gene among the tumor suppressor genes, with approximately 60% of human tumors presenting TP53 mutations. Current literature shows that OS exhibits a wide range of gene mutations and molecular alterations. However, apart from mutations in the TP53 and/or retinoblastoma (RB) genes, no novel gene mutations have been identified. Several studies have demonstrated that transfection of a functional copy of the TP53 gene could inhibit the growth of OS cells (Bekeredjian et al., 2007; Chen et al., 2012; Chen et al., 2010).

The introduction of exogenous genes relies on gene vectors, and the stability of the gene will be relatively poor without an optimal gene vector. In recent years, great progress has been made in vector research, and high transfection efficiencies have been shown for viral vectors. However, due to the high immunogenicity of viruses, their application is limited. Nonviral vectors such as liposomes are safe and less toxic compared with viral vectors, but instability and inefficiency remain constraints that cannot be ignored (Chertok et al., 2007; Du et al., 2011; Elzeny et al., 2017; Hida et al., 2011). Therefore, finding a safe, efficient, and targeted gene therapy transfection system has become the focus of researchers.

Poly (lactic-co-glycolic acid) (PLGA) nanobubbles (NBs) are nonviral vectors that offer significant advantages, including slow release, penetration, and ‘targeting’. These characteristics make PLGA NBs an ideal choice for drug or gene carriers (Huang et al., 2012; Hynynen et al., 2006; Jiang and Dalby, 2023). However, the “targeting” of this delivery system is still passive and depends on the free diffusion of the medium across the body. Superparamagnetic iron oxide (SPIO, diameter <10 nm) nanoparticles are a type of magnetic nanoparticle that are physically sensitive to external magnetic fields, and magnetic targeting (MT) can be applied to actively enhance the concentration in the target area (Lu et al., 2003; Sirsi et al., 2012). Hence, we hypothesized that the incorporation of SPIO and MT into TP53/PLGA NBs could serve as an effective strategy to augment gene transfection in the targeted area.

Recently, ultrasound (US)-mediated microbubble destruction (UTMD) has undergone rapid development. UTMD has been demonstrated to be a safe and effective method for gene transfection by numerous studies (Sun et al., 2023; Tang et al., 2012; Wells, 2010; Wu et al., 2020). UTMD has been successfully used for gene transfection of the kidney, testis, heart, pancreas, lung, skin, uterus, brain, retina diseases, and spinal cord (Sitta and Howard, 2021; Steiniger et al., 2004; Xenariou et al., 2007). Low-intensity-focused ultrasound (LIFU) can disrupt target nanobubbles at low intensity (referred to as relatively low-intensity mode, RLI mode), whereas at higher intensities (referred to as relatively high-intensity mode, RHI mode), LIFU induces range-controllable cytocidal effects on tissues (Yamaguchi et al., 2011; Yin et al., 2014; Zhong et al., 2012). To the best of our knowledge, few studies have investigated gene therapy for OS through US-magnetic-mediated NB destruction, and direct OS treatment using RHI-mode LIFU has not been reported. Thus, it remains unclear whether US-magnetic-mediated gene transfection in combination with LIFU represents an effective therapeutic strategy for OS.

In this study, we performed US-magnetic-mediated TP53-NB destruction and evaluated its potential to inhibit OS growth when combined with LIFU in both *in vitro* and *in vivo* experiments.

## Material and methods

### Preparations of SPIO/PLGA-NBs (SP-NBs), TP53/PLGA-NBs (TP-NBs) and SPIO/TP53/PLGA-NBs (STP-NBs)

NBs were freshly prepared prior to use. SPIO nanoparticles (mean diameter = 5 nm, concentration = 25 mg/mL) were purchased from Ocean Nanotech Co., Ltd. The TP53 plasmid was purchased from OriGene Technologies, Inc. and extracted using an endotoxin-free plasmid extract kit (enhanced) (DP120; TIANGEN BIOTECH (BEIJING) Co., Ltd.). PLGA (50:50, molecular weight [MW] = 12,000) was purchased from Polyscitech Co., Ltd. Polyvinyl alcohol (PVA, MW = 30,000-70,000) was purchased from Sigma Co., Ltd.

As reported previously, STP-NBs were prepared by the double-emulsion method. (Kiwada et al., 1986). First, 50 mg PLGA was completely dissolved in 2 mL dichloromethane, 600 µg TP53 plasmids and 20 µL SPIO were added to the solution. The mixture was emulsified in an ice bath using an ultrasonic processor for 5 min at a power of 175 W. Then, 5 mL cold PVA solution (4% W/V) was added to the initial emulsion, and an ultrasonic processor was used to further emulsify the product in the ice bath with a power of 125 W for 3 min. Then, 10 mL of 1% isopropanol solution was added to the second emulsion. The final solution was stirred with a magnetic agitator for 3 h. The volatilized solution was centrifuged at a low temperature centrifuge at 4°C for 10 min at 10000×g. The precipitate was washed with deionized water, and the supernatant was collected for further detection. The centrifugal washing step was repeated 3 times. Finally, the precipitate after washing was temporarily stored at 4°C for further use. SP-NBs and TP-NBs were similarly prepared without the addition of either TP53 or SPIO.

### Characterizations of TP-NBs and STP-NBS

A laser particle size analyzer system (Zeta SIZER, Malvern) was used to analyze the mean diameter, zeta potential, and polydispersity index (PDI) of the samples. Transmission electron microscopy (TEM; Hitachi H-7600) was used to evaluate the shape of the NBs, whereas scanning electron microscopy (SEM; JEOL JSM-7800F) was used to assess the morphological characteristics of the nanoparticles. (Zhao et al., 2015). The iron content of the STP-NBs was determined by the o-phenanthroline method. The encapsulation efficiency of SPIO is calculated as follows: encapsulation efficiency = W1/W2×100%, where W1 is the amount of SPIO in STP-NBs and W2 is the total amount of SPIO used to prepare STP-NBs. (Kiwada et al., 1986; Ting et al., 2012). We compared SPIO encapsulation efficiencies at five SPIO total inputs (0.1, 0.3, 0.5, 0.7 and 0.9 mg). The SPIO encapsulation efficiency test was repeated three times. The gene encapsulation efficiency was determined by an ultraviolet spectrophotometer (Thermo NanoDrop 2000; Thermo Fisher Scientific). The encapsulation efficiency of TP53 is calculated as follows: encapsulation efficiency = (W2-W1/W2 ×100)%, where W1 is the number of genes in the supernatant after centrifugation and W2 is the total amount of TP53 used to prepare STP-NBs. (Zhang et al., 2014; Wang et al., 2019). We compared TP53 encapsulation efficiencies at five TP53 plasmid total inputs (0.1, 0.2, 0.4, 0.8, and 1 mg). The TP53 encapsulation efficiency test was repeated three times. To assess gene release behavior, *in vitro* sonication was performed using an RLI-mode LIFU. The frequency was 0.95 MHz, the power was modulated at a pulse modulation of 300 Hz, and the depth of focus of the probe was 15 mm. The ultrasonic power is 1.5 W and lasts 60 s. STP-NBs (TP-NBs) from 10 mL phosphate buffered saline (PBS) were transferred to dialysis bags (molecular weight cut-off: 10,000 Da) and placed in a 100 mL PBS reservoir after ultrasound. After the appropriate time interval, 1 mL dialysate was taken, and the gene concentration was determined by ultraviolet spectrophotometry. One milliliter of fresh PBS was added to the reservoir to keep the liquid volume constant. The cumulative release ratio of TP53 released was calculated. To investigate the magnetization capacity, STP-NBs were placed under a magnetic field, and the adsorption time was recorded.

### *In vitro* studies on MG63 and HOS osteosarcoma cells

#### *In vitro* focused ultrasound (FUS) sonication setup

**FIGURE 1:**
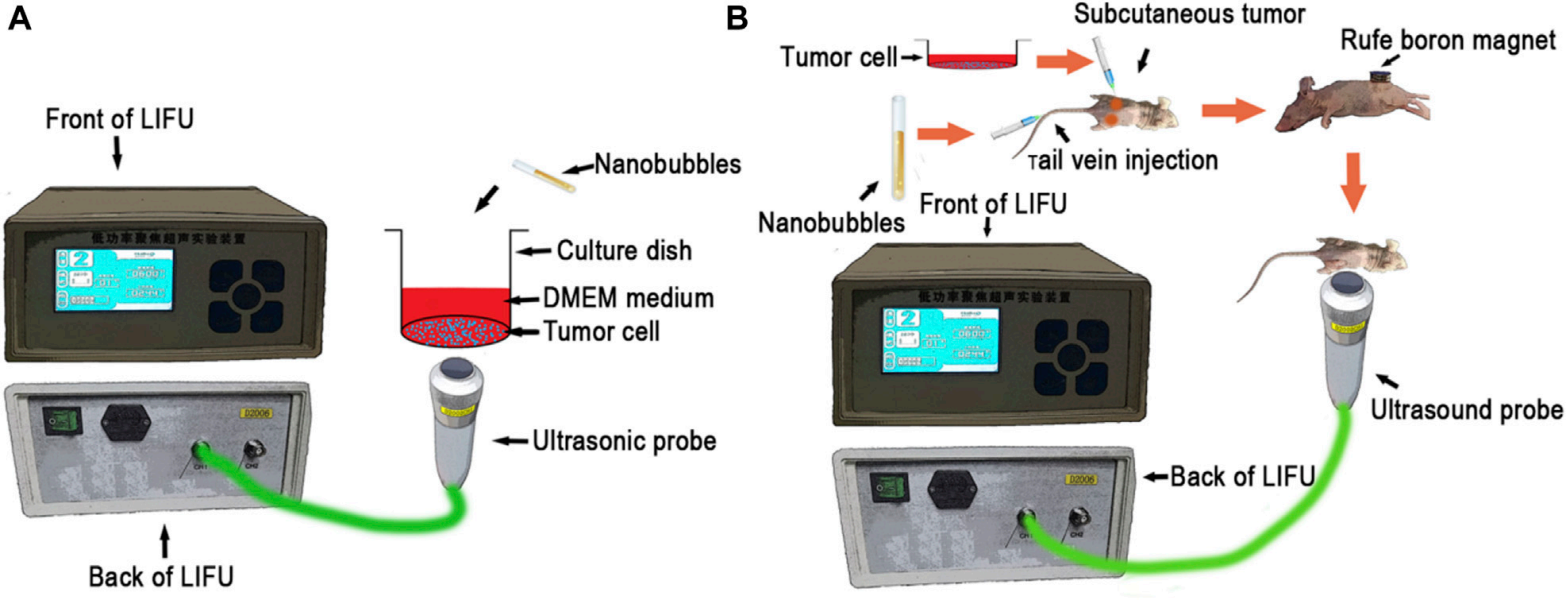
**(A)** Schematic of the *in vitro* experimental setup, **(B)** Schematic of the *in vivo* experimental setup.

The MG63 and HOS cell lines used in this study were acquired from Professor Tong-Chuan He (Chongqing Medical University, Chongqing, China). To assess gene release behavior, *in vitro* sonication was performed by using RLI-mode LIFU. The sonication parameters of RLI-mode were set at 1.5 W for 5 min (Figure 1A).

HOS and MG63 cells were randomly assigned into five treatment groups:

1. Control group: 50 µL PBS was added to 24-well plates, and 10 µL PBS was added to 96-well plates.
2. TP53 + SP-NBs group: 25 µL TP53 plasmid (1 µg) and 25 µL SP-NB solution were added to 24-well plates; 5 µL TP53 plasmid (0.2 µg) and 5 µL SP-NB solution were added to 96-well plates.
3. TP53 + US group: 25 µL TP53 plasmid (1 µg) and 25 µL PBS solution were added to 24-well plates, which were then subjected to RLI-mode LIFU irradiation; 5 µL TP53 plasmid (0.2 µg) and 5 µL PBS solution were added to 96-well plates, which were then subjected to RLI-mode LIFU irradiation (Figure 1A).
4. TP-NBs + US group: 50 µL TP-NB (TP53, 1 µg) solution was added to 24-well plates, which were then subjected to RLI-mode LIFU irradiation; 10 µL TP-NB (TP53, 0.2 µg) solution was added to 96-well plates, which were then subjected to RLI-mode LIFU irradiation (Figure 1A).
5. STP-NBs + US group: 50 µL STP-NB (TP53, 1 µg) solution was added to 24-well plates, which were then subjected to RLI-mode LIFU irradiation; 10 µL STP-NB (TP53, 0.2 µg) solution was added to 96-well plates, which were then subjected to RLI-mode LIFU irradiation (Figure 1A).

### Cell proliferation assay

The proliferative capacity of cells was detected using a cell counting kit-8 assay (CCK8, MedChemExpress, United States). MG63 and HOS cells were seeded in 96-well plates at a density of 2000 cells/well and cultured for 36 h. Then, the cells were grouped, treated as specified above and cultured for 2 h. After that, the cells were washed three times with PBS and cultured for another 36 h in fresh cell culture medium. Finally, 10 µL CCK8 reagent was added to the wells. After the plates were incubated for 0.5–4 h, cell proliferation was detected by measuring the absorbance at 450 nm with a microplate reader. Cell viability (%) relative to that of the control cells was calculated according to the instructions of the CCK-8 assay. (Livak and Schmittgen, 2001).

### Real-time PCR

MG63 and HOS cells were seeded in 24-well plates at a density of 1*105 cells/well and cultured for 36 h. Then, the cells were grouped, treated as specified above and cultured for 2 h. After that, the cells were washed three times with PBS and cultured for another 36 h in fresh cell culture medium. Finally, total RNA was isolated by using TRIzol reagent (TAKARA BIO INC) following the manufacturer’s protocols. The PrimeScript™ RT reagent kit was used to reverse transcribe RNA to complementary DNA (cDNA). SYBR Green Super Mixture (TAKARA BIO INC) was used to amplify the resulting cDNA. The P53 primer sequences were as follows: 5′- GCC​ATC​TAC​AAG​CAG​TCA​CAG​C-3′ (sense) and 5′- CAT​CCA​AAT​ACT​CCA​CAC​GCA​A-3′ (antisense). The GAPDH primer sequences were as follows: 5′-TCA​AGA​AGG​TGG​TGA​AGC​AGG-3′ (sense) and 5′-AGC​GTC​AAA​GGT​GGA​GGA​GTG-3′ (antisense). The CFX-Connect real-time PCR system (Bio-Rad) was used to conduct qRT-PCR in 96-well plates. The amplification conditions were as follows: 95°C for 3 min, followed by 45 cycles of 95°C for 10 s and 58°C for 30 s. Each qRT-PCR analysis was carried out in triplicate, and the 2^−ΔΔCT^ method was used to analyze the data.

### Western blotting analysis

MG63 and HOS cells were seeded in 24-well plates at a density of 1*105 cells/well and cultured for 36 h. Then, the cells were grouped, treated as specified above and cultured for another 2 h. After that, the cells were washed three times with PBS and cultured for an additional 36 h in fresh cell culture medium. Finally, proteins were homogenized in lysis buffer and phenylmethanesulfonyl fluoride (Beyotime, China). The bicinchoninic acid assay (Beyotime Institute of Biotechnology, China) was used to determine the protein concentrations. The lysates were centrifuged at a low temperature centrifuge at 4°C for 10 min at 10000×g, and the supernatants were collected and transferred into separate enzyme-free tubes. The same amounts of protein were separated by SDS-PAGE and transferred to a polyvinylidene fluoride (PVDF) membrane. The membranes were blocked in 5% skim milk for 1 h at room temperature and incubated with specific primary antibodies at 4°C overnight. The membranes were sealed in 5% skim milk at room temperature for 1 h and incubated with specific primary antibody (anti-P53, Anffinity, United States; 1:1,000; anti-GAPDH, Anffinity, United States; 1:10000) overnight at 4°C. After washing with TBST three times, membranes were incubated with the secondary antibodies at room temperature for 1 h. An Immobilon Western Chemiluminescent Kit (NCM Biotech, China) was used to visualize the labeled protein bands. The band density of P53 protein was normalized to GAPDH, and all western blots were quantified using ImageJ software.

### In vivo studies on osteosarcoma

#### *In vivo* experimental setup

This study was conducted according to the guidelines for the care and use of experimental animals from the National Institutes of Health, and the experimental schemes were approved by the Animal Ethics Committee of Chongqing Medical University (Figure 1B).

HOS cells were digested and collected at 90% density and resuspended in serum-free 1,064 medium to 5 × 10^7^ cells/mL. Each athymic nude mouse (4 weeks old, Beijing HFK Bioscience Corporation, China) was subcutaneously injected with 100 μL cell suspension (approximately 5 × 10^6^ cells). (Lin et al., 2019). To assess gene release behavior, *in vitro* sonication was performed by using LIFU. The sonication parameters of the RLI-mode were 1.5 W for 5 min, and the parameters of the RHI-mode were set as follows: power, 12 W; sound intensity, 19.2 w/cm2 at focus; depth of focus of 1.5 cm. Two weeks after subcutaneous implantation, the tumor-bearing nude mice were randomly assigned into seven treatment groups:

1. Control group: 1 mL NS was injected via the tail vein.
2. TP53 + SP-NBs + MT group: 0.5 mL TP53 plasmid (50 µg) and 0.5 mL SP-NB solution were injected via the tail vein. For animals undergoing the MT procedure, a permanent magnet was placed tightly to the surface skin of the tumor nodules for 30 min.
3. TP53 + US group: 0.5 mL TP53 plasmid (50 µg) solution and 0.5 mL NS were injected via the tail vein and subjected to RLI-mode LIFU irradiation.
4. TP-NBs + MT + US group: 1 mL TP-NB (TP53, 50 µg) solution was injected via the tail vein, and a RuFe boron magnet was applied tightly to the surface skin of the tumor nodules for 30 min before the mice were subjected to RLI-mode LIFU irradiation (Figure 1B).
5. STP-NBs + US group: A 1 mL STP-NB (TP53, 50 µg) solution was injected via the tail vein, and then the mice were subjected to RLI-mode LIFU irradiation.
6. STP-NBs + US + MT group: A 1 mL STP-NB (TP53, 50 µg) solution was injected via the tail vein, and then a permanent magnet was placed tightly to the surface skin overlaying the tumor nodules for 30 min before the mice were subjected to RLI-mode LIFU irradiation (Figure 1B).
7. STP-NBs + US + MT + LIFU group: A 1 mL STP-NB (TP53, 50 µg) solution was injected via the tail vein, and then a RuFe boron magnet was applied tightly to the surface skin of the tumor nodules for 30 min. Next, the mice were subjected to RLI-mode LIFU irradiation followed by RHI-mode LIFU irradiation for 10 min (Figure 1B).

All mice received their respective drug injections every 24 h, which was continued for 5 days, and tumor nodules were collected 7 days after the last transfection. The retrieved tumor tissues were fixed in 4% paraformaldehyde for 24 h and embedded in paraffin. During the treatment, all nude mice were intraperitoneally injected with pentobarbital for deep anesthesia.

### Perls’ Prussian blue assay

The iron distribution was determined by examining tissue sections stained with Perls’ Prussian blue by light microscopy. (Asano et al., 2006). Briefly, the tissue sections were dewaxed with xylene, rehydrated with reduced ethanol concentration, rinsed with distilled water, and incubated with the staining solution for 20 min. Then, the tissue sections were rinsed with distilled water for 5 min and counterstained with nuclear fast red for 5 min. Prussian blue-positive cells were stained blue. Sections were imaged under a microscope, and the number of positive cells was counted.

### TUNEL assay

A terminal deoxynucleotidyl transferase-mediated dUTP-biotin nick end labeling (TUNEL) staining kit was used to analyze OS apoptosis. Briefly, tissue sections were dewaxed in xylene, rehydrated by reducing the concentration of ethanol, washed in distilled water, protease treated, and incubated with TUNEL reactive mixture. Nuclear reverse staining with 4′,6-diamidino-2-phenylindole (DAPI). The nuclei of TUNEL-positive cells were dense and showed green fluorescence, suggesting apoptosis. The sections were imaged by fluorescence microscopy.

### Total TP53 expression *in vivo* after transfection

Quantification of gene expression was determined by qRT-PCR as previously described. TP53 protein expression was measured using immunohistochemistry. (Mungarndee et al., 2008). Briefly, tumor tissue sections were incubated at 4°C overnight with a primary antibody and then incubated at room temperature for 1 h with a secondary antibody. The expression of target proteins was observed by using chromogenic DAB substrate. Sections were imaged under a microscope.

### Statistical analysis

SPSS 21 software (IBM, Armonk, NY, United States) was used for statistical analysis. All data are presented as the mean ± standard error of the mean of at least three independent samples. The differences among multiple groups were determined by using one-way ANOVA (*p* < 0.05). The differences between two groups at the same time point were analyzed by using Student’s t-test. A *p*-value of less than 0.05 indicated statistical significance.

## Results

### TP-NBs and STP-NBs were well prepared and with excellent functional characteristics

**FIGURE 2:**
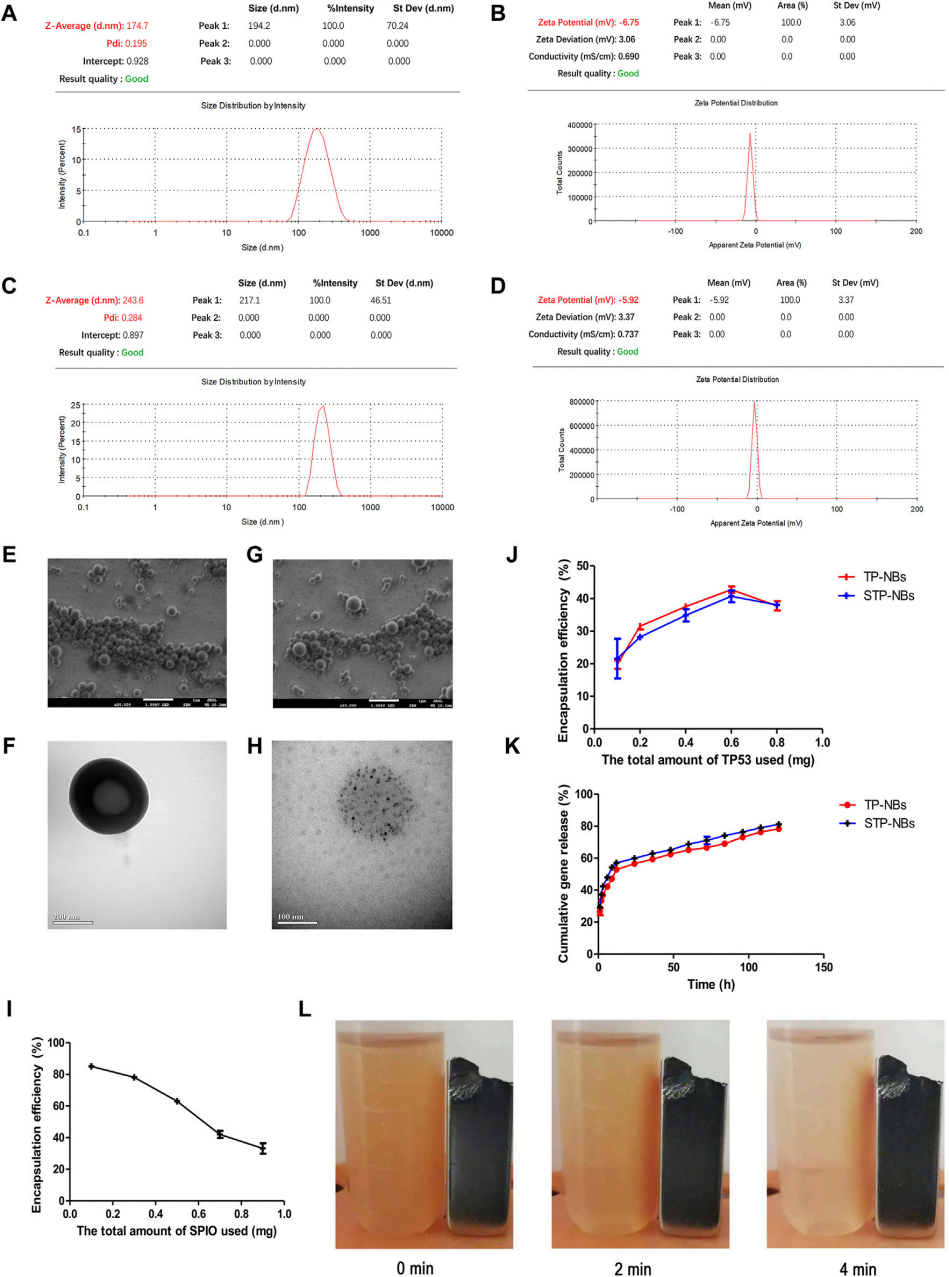
Characterization of PP-NBs and SPP-NBS NBs. **(A)** The mean diameter of TP-NBs, **(B)** The zeta potential of TP-NBs, **(C)** The mean diameter of STP-NBs, **(D)** The zeta potential of STP-NBs, **(E)** SEM of TP-NBs, **(F)** TEM of TP-NBs, **(G)** SEM of STP-NBs, **(H)** TEM of STP-NBs, **(I)** SPIO encapsulation efficiency of STP-NBs, **(J)** TP53 plasmid encapsulation efficiency of TP-NBs and STP-NBs, **(K)** Cumulative gene release of TP-NBs and STP-NBs, **(L)** Suspended STP-NBs were attracted to the permanent magnet.

The TP-NB zeta potential was −6.75 ± 3.06 mV, the mean diameter was 174.7 ± 70.24 nm, and the PDI was 0.195 (Figures 2A, B). The STP-NB zeta potential was −5.92 ± 3.37 mV, the mean diameter was 243.6 ± 46.51 nm, and the PDI was 0.284 (Figures 2C, D). SEM and TEM analysis revealed that TP-NBs and STP-NBs were highly dispersed and had a well-defined spherical morphology, and SPIO was observed in the core of STP-NBs (Figures 2E–H). The o-phenanthroline method was used to measure the SPIO encapsulation efficiency of STP-NBs, and the SPIO encapsulation efficiency decreased with increasing total amount of SPIO. The results revealed that the SPIO content was highest when 0.5 mg of SPIO was used for encapsulation (Figure 2I). The ultraviolet spectrophotometry method was used to measure the TP53 plasmid encapsulation efficiency of TP-NBs and STP-NBs, and when 0.6 mg of TP53 plasmid was used, the TP53 plasmid encapsulation efficiency was highest (Figure 2J). The ultraviolet spectrophotometry method was used to measure the release efficiency of TP53 from TP-NBs and STP-NBs after ultrasonic irradiation. A total of 57.1% of TP53 was released from STP-NBs within 12 h after ultrasonic irradiation; 65.1% of TP53 was released at 48 h after ultrasonic irradiation. Regarding STP-NBs, 52.9% of the loaded TP53 was released from STP-NBs at 12 h after ultrasonic irradiation, and 62.4% was released at 48 h (Figure 2K). In addition, the suspended STP-NBs were attracted by permanent magnet when placed under a magnetic field for 4 min, indicating that STP-NBs have a high magnetization ability (Figure 2L).

### Ultrasound-targeted transfection of TP53 in two kinds of OS cell lines and the overexpression of P53, resulting in efficient inhibition of OS cells proliferation *in vitro* studies

**FIGURE 3:**
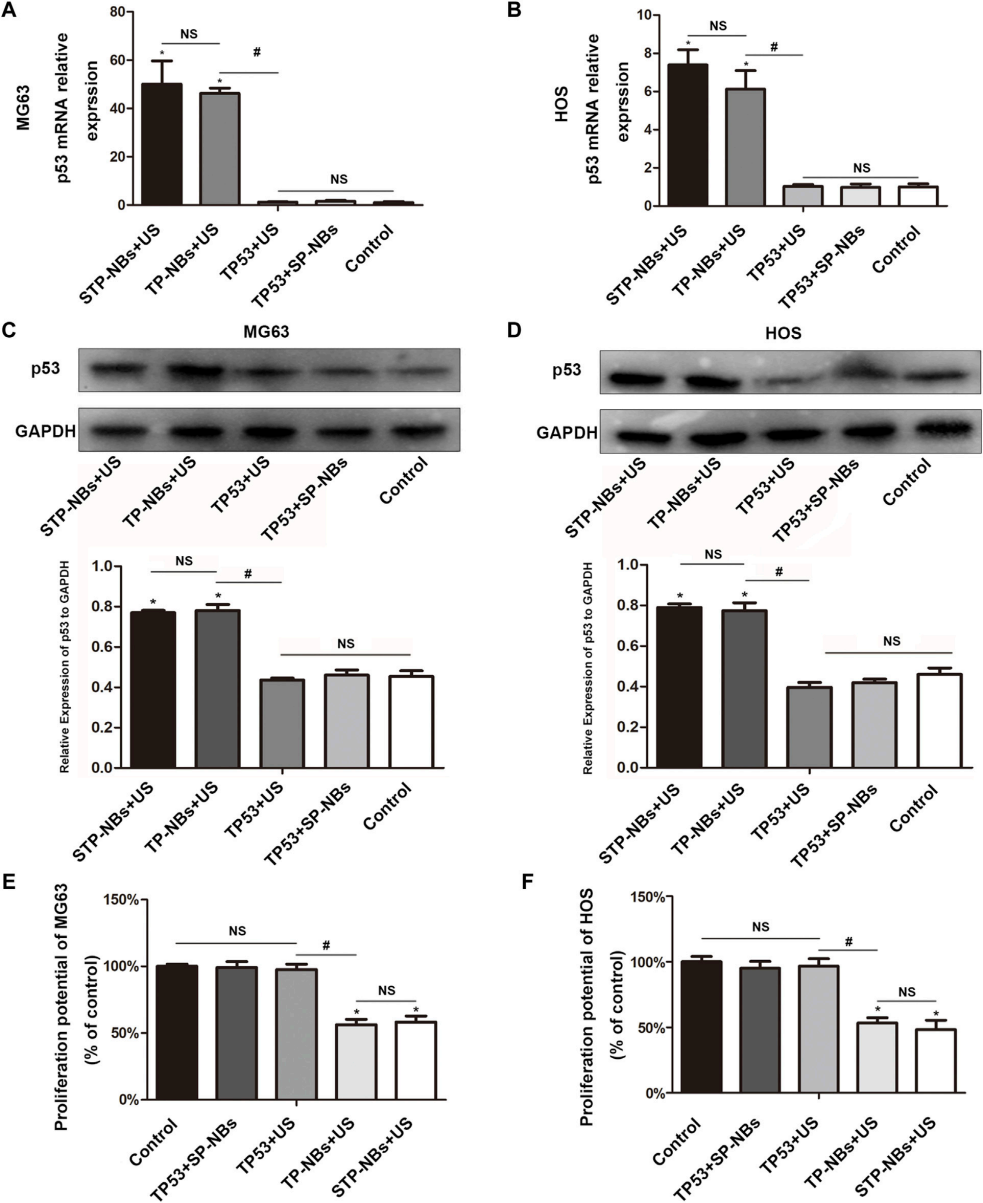
**(A)** qRT-PCR of MG63 cells, **(B)** qRT-PCR of HOS cells, **(C)** WB of MG63 cells, **(D)** WB of HOS cells, **(E)** Proliferation potential of MG63 cells, **(F)** proliferation potential of HOS cells. *, *p* < 0.05, compared with the Control group; NS = no significance; #, *p* < 0.05, compared with the TP53 + US group.

The 2 -ΔΔCT method was used to analyze the qRT-PCR data. () TP53 mRNA expression in MG63 cells was significantly higher in the TP-NBs + US and STP-NBs + US groups than in the other groups at 36 h after transfection (*p* < 0.05) (Figure 3A). Furthermore, the expression patterns were not significantly different between the TP-NBs + US and STP-NBs + US groups. and were also not significantly different between the control group, TP53 + SP-NBs group and TP53 + US group (*p* > 0.05) (Figure 3A). Western blotting (WB) was used to detect TP53 protein expression. A double band was observed for each group. TP53 protein expression in MG63 cells 36 h after treatment was highest in the TP-NBs + US and STP-NBs + US groups (Figure 3C). There was no significant difference between the TP-NBs + US and STP-NBs + US groups (*p* > 0.05) and was also not significantly different among the control group, TP53 + SP-NBs group and TP53 + US group (*p* > 0.05) (Figure 3C). Similar results were shown for HOS cells (Figures 3B, D).

The proliferation potential of MG63 cells was detected 36 h after treatment. MG63 cell activity in the TP-NBs + US group and STP-NBs + US group decreased 36 h compared with the other groups (*p* < 0.05). There was no significant difference in proliferation rates between the TP-NBs + US group and the STP-NBs + US group (*p* > 0.05), and there was also no significant difference among the control group, TP53 + STP-NBs group and TP53 + US group (*p* > 0.05) (Figure 3E). Similar results were shown for HOS cells (Figure 3F). So the proliferation of OS cells was efficiently inhibited by the overexpression of TP53 *in vitro*.

### Ultrasound-targeted transfection in magnetic field enhanced the transfection efficiency of the TP53 plasmid in local area, resulting in apoptosis on the osteosarcoma model *in vivo*

**FIGURE 4:**
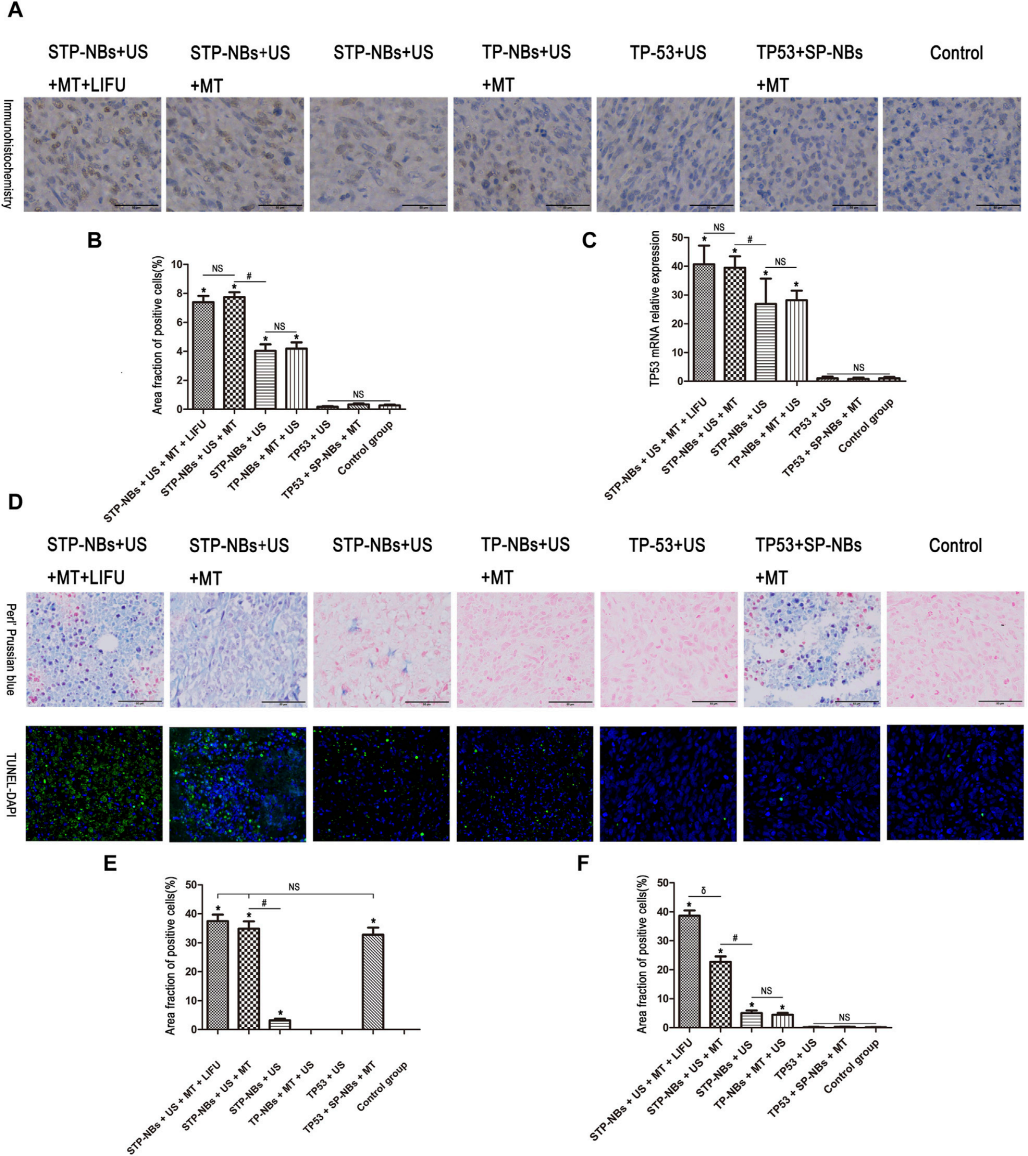
**(A)** Statistical chart of TUNEL-DAPI staining, **(B)** Statistical chart of immunohistochemistry, **(C)** Statistical chart of qRT-PCR. **(D)** Section staining of osteosarcoma tissue for Perls’ Prussian blue and the TUNEL assay, **(E)** Statistical chart of Perls’ Prussian blue assay, **(F)** Statistical chart of the TUNEL assay. *, *p* < 0.05, compared with the Control group; NS = no significance; #, *p* < 0.05, compared with the STP-NBs group; δ, *p* < 0.05, compared with the STP-NBs + US + MT group.

TP53 protein expression was detected by immunohistochemistry *in vivo* and showed increases in groups 4, 5, 6 and 7 compared with the other three groups (Figures 4A, B). Groups 6 and 7 showed the highest TP53 protein expression, but there was no significant difference (*p* > 0.05) between the two groups. In addition, there was also no significant difference in TP53 expression between groups 4 and 5(*p* > 0.05). Groups 1, 2 and 3 showed the least TP53 protein expression, the values of which were not significantly different among them (*p* > 0.05) (Figures 4A, B). The 2 -ΔΔCT method was used to analyze the qRT-PCR data. TP53 mRNA expression was increased in groups 4, 5, 6 and 7 compared with that in the other three groups (Figure 4C). Groups 6 and 7 showed the highest TP53 mRNA expression, but there was no significant difference (*p* > 0.05) between the two groups, and there was also no significant difference between groups 4 and 5 (*p* > 0.05). Groups 1, 2 and 3 showed the lowest TP53 mRNA expression, which was not significantly different among them (*p* > 0.05) (Figure 4C).

The iron distribution was detected using Perls’ Prussian blue assay 7 days after transfection, and the results showed that the iron content was significantly higher in groups 2, 6 and 7 than in the other groups (*p* < 0.05); furthermore, these three groups exhibited no significant differences (*p* > 0.05) (Figures 4D, E). In addition, group 5 showed the highest iron content among all the treatment groups. There was also no significant difference among groups 1, 3, and 4(*p* > 0.05) (Figures 4D, E).

The TUNEL assay was used to measure OS apoptosis. The TUNEL-DAPI assay showed significantly increased apoptosis in groups 4, 5, 6 and 7 and significantly higher apoptosis than the other three groups (*p* < 0.05) (Figures 4D, F). Group 7 showed the highest number of TUNEL-negative cells, followed by group 6 (*p* < 0.05). The degree of apoptosis in groups 4 and 5 was not significantly different (*p* > 0.05) (Figures 4D, F). A few TUNEL-negative cells were found in groups 1/2/3 and showed no significance (*p* > 0.05) (Figures 4D, F). So these results demonstrated that ultrasound-targeted transfection in magnetic field enhanced the transfection efficiency of the TP53 plasmid and the overexpression of TP53 in local area, resulting in apoptosis on the osteosarcoma model *in vivo*.

## Discussion

Current clinical treatment modalities for OS include surgical resection of primary tumors and systemic chemotherapy. (Panez-Toro et al., 2023). In recent years, the therapeutic use of exogenous genes for treating OS has gained much interest. TP53 is one of the most well-studied tumor suppressor genes. Previous studies have shown that gene transduction of functional TP53 can effectively inhibit OS cell activity. The purpose of gene therapy is to correct abnormal protein expression at the gene level, and the introduction of exogenous TP53 to correct the reduction of TP53 level is a promising OS therapy method. (Tsuchiya et al., 2000; Ganjavi et al., 2006). The mechanism of TP53 is well understood in OS. (Song et al., 2015).

Magnetic nanoparticles could become magnetized and exhibit physical sensitivity to external magnetic fields. Thus, it has been considered to lead to an increase in the local drug concentration at the target site. It was initially used merely for enhancing magnetic resonance imaging in most of the current studies. (Kiwada et al., 1986; Liu et al., 2009; Ting et al., 2012). However, these characteristics signify that magnetic nanoparticles are one kind of most suitable material for gene therapies.

Ultrasound irradiation is an ideal physical gene transfection method to promote extracellular molecules to enter cells. (Feril, 2009). The LIFU device used in this study was developed and patented by our institute. Current studies have indicated that RLI-mode LIFU can destroy target nanobubbles, which not only does not destroy exogenous genes but also improves transfection efficiency; meanwhile, increasing the intensity (RHI-mode) of the LIFU device would exert range-controllable cytocidal effects on tissues. (Xu et al., 2009; Song et al., 2017). To date, limited research on the gene therapy of OS by ultrasound-magnetic-mediated NB destruction has been conducted, and OS treatment using RHI-mode LIFU directly has not been reported.

In an *in vitro* study, the results provide evidence of the successful preparation of STP-NBs. They had a mean diameter of 243.6 ± 46.51 nm, a PDI of 0.284, and a zeta potential of −5.92 ± 3.37 mV. The optimal total input of SPIO and TP53 plasmids was 0.5 mg and 0.6 mg, respectively. The STP-NBs showed good sustained release of genes. In addition, the physical sensitivity of STP-NBs to external magnetic fields has been proven *in vitro*.

In the cell experiments, we loaded STP-NBs with TP53 and administered RLI-mode LIFU to treat MG63 and HOS cells. TP53 protein and mRNA expression in cells were not significantly different between the TP-NBs + US and STP-NBs + US groups. The CCK8 results showed that cell activity was decreased in the TP-NBs + US and STP-NBs + US groups, but there was no significant difference between them. The TP53 + SP-NBs group was not significantly different from the control group for the TP53 + US group. Furthermore, it was also shown that STP-NBs have no cytotoxicity. These results suggest that STP-NBs were able to transfect MG63 cells and HOS cells. The TP53 plasmid was well expressed and inhibited cell proliferation.

In the animal experiments, we used both STP-NBs loaded with TP53 and RHL-mode LIFU to treat tumor-bearing nude mice. The Perls’ Prussian blue assay showed that the iron content was significantly higher in groups 2, 6 and 7 than in the other groups, but no significant difference was observed among these three groups. This suggests that STP-NBs can be concentrated in the targeting area in response to external magnetic fields *in vivo*.

The protein and mRNA expression levels of TP53 were increased in groups 4, 5, 6 and 7 compared with the other three groups. Groups 6 and 7 showed the highest TP53 expression, but there was no significant difference between these two groups; in addition, there was also no significant difference between groups 4 and 5. Groups 1, two and three showed the least TP53 expression, and there was no significant difference between them. These data indicate that STP-NBs were able to mediate TP53 transfection and expression, and the external magnetic field improved the transfection efficiency of STP-NBs *in vivo*.

The TUNEL assay showed significantly increased apoptosis in groups 4, 5, 6 and 7 compared with that of the other three groups. Group 7 showed the highest number of giant, disorganized, TUNEL-negative structures with large clusters of nuclei; group 6 had the next highest map. The degree of apoptosis in groups 4 and 5 was not significantly different. A few TUNEL-negative cells were found in groups 1, 2 and 3, and there was no observed statistical significance. The results demonstrated that following transfection and expression of the TP53 gene, the rate of apoptosis in OS tissue was increased *in vivo*. Meanwhile, this effect further progressed the addition of external magnetic fields and RHI-mode LIFU *in vivo* to our testing ro.

When interpreting these findings, several limitations must be considered. The best guidance method is an external focusing magnetic field located in the center of the tumor body. However, this study was limited by the available equipment, and all magnetic field guidance was generated by the RuFe boron magnet on the surface of the tumor. The strength of the magnetic field in different parts of the tumor was varied. A convergent magnetic field device that can accurately locate the focus will improve future studies of gene therapy. SPIO could enhance magnetic resonance imaging, which could potentially better demonstrate the concentration of nanoparticles in animals. (Fan et al., 2016). Due to equipment limitations, that study could not proceed. Furthermore, the LIFU waves used in this study may have released TP53 into other organs, causing additional biological effects. Previous studies only demonstrated the feasibility of the combination of LIFU and ultrasound-magnetic-mediated Spio-P53-nanobubble destruction at the cell and organism levels. (Elumalai et al., 2024). As a kind of high capacity of reflection biological tissues, less ultrasound energy is able to penetrate the inner portion of the bony tissue. Although high-intensity focused ultrasound (HIFU) experiments have proven that sound attenuation decreases and the sound beam easily passes through because of OS bone destruction, (Wu et al., 2001), the ossifying tumor of the human body needs further study. However, despite these limitations, this does not prevent us from trying some new research methods to treat OS more effectively or to exlpore new method to prepare for surgery.

## Conclusion

We used LIFU combined with US-magnetic-mediated SPIO-TP53-NB destruction for OS treatment. Our findings indicate that the destruction of US-magnetic-mediated SPIO/P53/PLGA/NBs facilitates gene transfection and that LIFU combined with the destruction of these NBs may represent a novel, noninvasive and targeted therapy for OS.

## Figures and Tables

**Table:** 

**CCK8**	cell counting kit-8 assay
**DAB**	diaminobenzidine
**DAPI**	4′,6-diamidino-2-phenylindole
**DMEM**	Dulbecco’s modified Eagle medium
**FBS**	fetal bovine serum
**FUS**	focused ultrasound
**HIFU**	high-intensity-focused ultrasound
**LIFU**	low-intensity-focused ultrasound
**MT**	magnetic targeting
**MW**	molecular weight
**NBs**	nanobubbles
**NS**	normal saline
**OS**	osteosarcoma
**PBS**	phosphate-buffered saline
**PDI**	polydispersity index
**PLGA**	poly lactic-coglycolic acid
**PVA**	polyvinyl alcohol
**PVDF**	polyvinylidene fluoride
**qRT-PCR**	quantitative reverse transcription polymerase chain reaction
**RB**	retinoblastoma
**RLI-mode**	relatively low-intensity mode
**RHI-mode**	relatively high-intensity mode
**SEM**	scanning electron microscope
**SPIO**	superparamagnetic iron oxide
**SP-NBs**	superparamagnetic iron oxide/poly lactic-co-glycolic acid nanobubbles
**STP-NBs**	superparamagnetic iron oxide/TP53/poly lactic-co-glycolic acid nanobubbles
**TEM**	transmission electron microscopy
**TP-NBs**	TP53/poly lactic-co-glycolic acid nanobubbles
**US**	ultrasound
**UTMD**	ultrasound-mediated microbubble destruction
**WB**	Western blotting

## Data Availability

The raw data supporting the conclusion of this article will be made available by the authors, without undue reservation.
